# EVOO’s Effects on Incretin Production: Is There a Rationale for a Combination in T2DM Therapy?

**DOI:** 10.3390/ijms231710120

**Published:** 2022-09-04

**Authors:** Simona Amodeo, Luigi Mirarchi, Aurelio Seidita, Roberto Citarrella, Anna Licata, Maurizio Soresi, Juan Lucio Iovanna, Lydia Giannitrapani

**Affiliations:** 1 Department of Health Promotion Sciences, Maternal and Infant Care, Internal Medicine and Medical Specialties (PROMISE), University of Palermo, 90127 Palermo, Italy; 2 Centre de Recherche en Cancérologie de Marseille, INSERM U1068, CNRS UMR 7258, Aix-Marseille Université and Institut Paoli-Calmettes, Parc Scientifique et Technologique de Luminy, F-13288 Marseille, France; 3 Institute for Biomedical Research and Innovation (IRIB), National Research Council, Via U. La Malfa 153, 90146 Palermo, Italy

**Keywords:** EVOO, GLP-1, MedDiet, T2DM

## Abstract

Type 2 diabetes mellitus (T2DM) is a serious public health concern as it is one of the most common chronic diseases worldwide due to social and economic developments that have led to unhealthy lifestyles, with a considerable impact both in terms of morbidity and mortality. The management of T2DM, before starting specific therapies, includes cornerstones such as healthy eating, regular exercise and weight loss. Strict adherence to the Mediterranean diet (MedDiet) has been related to an inverse association with the risk of T2DM onset, as well as an improvement in glycaemic control; in particular, thanks to the consumption of extra virgin olive oil (EVOO). Agonists of gut-derived glucagon-like peptide-1 (GLP-1), gastrointestinal hormones able to increase insulin secretion in response to hyperglycaemia (incretins), have been recently introduced in T2DM therapy, quickly entering the international guidelines. Recent studies have linked the action of EVOO in reducing postprandial glycaemia to the increase in GLP-1 and the reduction of its inactivating protease, dipeptidyl peptidase-4 (DPP-4). In this review, we explore observations regarding the pathophysiological basis of the existence of an enhanced effect between the action of EVOO and incretins and, consequently, try to understand whether there is a rationale for their use in combination for T2DM therapy.

## 1. Introduction

Type 2 diabetes mellitus (T2DM) is one of the most common chronic diseases worldwide. According to the 2021 report of the International Diabetes Federation, there are estimated to be around 537 million adults with T2DM between the ages of 20 and 79, and this number is expected to increase to 643 million in 2030 and to 783 million in 2045, an increase of 46% [1].

Diabetes is a disabling disease associated with a poor prognosis. In fact, it is estimated that, in 2021, 6.7 million adults aged 20–79 died from diabetes and/or its complications. This corresponds to 12.2% of global deaths from all causes in this age group. In addition, about one third (32.6%) of these deaths occurred before the age of 60 [1].

The development of T2DM is closely related to the loss of the functional mass of beta cells. Consequently, the maintenance or regeneration of beta cells should be strongly recommended as the ideal treatment for this condition. Indeed, the goal of therapy should be to stop the loss of beta cells and/or promote the restoration of fully functional beta cell mass [2].

Diabetes is also a major cause of morbidity. Among diabetes-related complications, atherosclerotic cardiovascular disease (ASCVD) remains the leading cause of mortality [3]. Events of ASCVD in diabetes mellitus include coronary artery disease, ischemic stroke, peripheral artery disease (PAD) and heart failure [4]. Controlling the main cardiovascular disease risk factors (HbA1c, LDL, blood pressure, albuminuria, cigarette smoking) in diabetic patients has allowed a reduction in the incidence of these complications, which are, however, still higher than in the non-diabetic population [5,6,7].

It is therefore clear that secondary prevention therapies, while effective, are unable to bridge the gap between diabetic and non-diabetic patients, despite optimal glycaemic control [8,9,10].

Among the therapies recently introduced for treating T2DM, GLP-1 receptor agonists have been extensively studied because they stimulate insulin secretion and reduce glucagon secretion in a glucose-dependent manner, improving satiety and promoting weight loss [11,12].

According to the recommendations of the American Diabetes Association and the European Association for the Study of Diabetes [13], lifestyle interventions, such as adopting a healthy diet and physical activity, are effective and safe for improving glycaemic control in T2DM and are recommended as first-line therapies from the time of diagnosis. A further contribution, therefore, could be given by the Mediterranean diet (MedDiet); in fact, recent meta-analyses have shown significant inverse associations between a high adherence to the MedDiet and the risk of T2DM onset, as well as an improvement in glycaemic control [14,15]. In addition, the consumption of extra virgin olive oil (EVOO) could have beneficial effects in the prevention, development and progression of T2DM [16].

Recent meta-analyses of randomized controlled trials (RCTs) have consistently shown that replacing carbohydrates (5–10% of total energy intake) with monounsaturated fatty acids (MUFA) as a specific dietary compound has beneficial effects on metabolic risk factors in patients with T2DM [17,18].

In this review, we focus on evidence of a link between EVOO consumption with the MedDiet and incretin production in order to understand if there is a rationale for their combination in T2DM therapy.

## 2. Glucagon-like Peptide-1 Receptor Agonists (GLP-1RAs)

Gut-derived glucagon-like peptide-1 (GLP-1) belongs to the family of incretin hormones, gastrointestinal hormones released after nutrient intake with the ability to increase insulin secretion in a glucose-dependent manner through pancreatic beta cells during periods of hyperglycaemia. They also stimulate the proliferation and neogenesis of beta cells, reduce their apoptosis and inhibit glucagon secretion from pancreatic alpha cells. The action of incretins can be influenced by several nutrients, mainly glucose and carbohydrates [19]. Their concentrations are low during fasting but rapidly increase after the ingestion of food.

The discovery of GLP-1 and its physiological functions triggered the research and development of GLP-1 receptor agonists (GLP-1 RAs) [20].

Initial studies in mouse models indicated that GLP-1 is highly effective as an insulinotropic agent in non-diabetic and metabolically healthy animals, while exhibiting substantially reduced biological activity in diabetic animals together with the insulinotropic polypeptide (GIP) [21]. This activity was subsequently also demonstrated in diabetic human subjects; however, GLP-1 degrades rapidly and is inactivated by the action of dipeptidyl peptidase-4 (DPP-4) protease, with a half-life of about 2 min [22]. GLP-1 RAs affect the endogenous incretin hormone, GLP-1 [23].

GLP-1 receptor is synthesized in the pancreatic islets, kidneys, lungs, heart and nervous system. After GLP-1 and GLP-1 RAs bind to the receptor, glucose-dependent insulin secretion is induced [24], thus rendering the stimulation of the GLP-1 receptor a suitable method for reducing plasma glucose in subjects with T2DM. The beneficial effects derived from the stimulation of the GLP-1 receptor act through three main mechanisms: by increasing glucose-dependent insulin secretion, reducing glucagon secretion and reducing gastric emptying speed with a consequent reduction in postprandial glycaemic excursions [22,25,26]. Other important beneficial effects, such as reducing appetite and, consequently, food consumption, resulting in a reduction in caloric intake and body weight, have also been demonstrated [27]. Based on this knowledge, research in this area identified the exendin-4 peptide in the saliva of a venomous lizard (*Heloderma suspectum*, the Gila monster) homologous to mammalian GLP-1 and able to bind and activate GLP-1 receptors [28,29]. Synthetic exendin-4 was called exenatide and was the first GLP-1 receptor agonist approved for the treatment of T2DM. The GLP-1 RA class offers important advantages in the treatment of T2DM.

All agents within this class not only demonstrate significant reductions in HbA1c but also a favourable effect on weight and a lower risk of hypoglycaemia. Treatment with GLP-1 receptor agonists is also recommended as a first option in patients with T2DM at onset, especially in those at risk of cardiovascular disease, due to an important effect on both the primary and secondary prevention of ischemic disease [30]. In particular, liraglutide, dulaglutide and semaglutide have been shown to reduce major cardiovascular events.

Paradoxically, the therapeutic strategies used for intensive glycaemic control have failed to be proven useful in the prevention of major adverse cardiovascular events (MACE) compared to less rigorous strategies [23], while the use of GLP-1RAs, which, in addition to affecting HbA1c, act on blood pressure and kidney damage, offers cardiovascular protection independent of glycaemic control.

To date, 14 direct head-to-head studies among GLP-1 RAs have been published. These studies confirm that there are differences among the various molecules belonging to this class, both in terms of their effectiveness in reducing HbA1c and body weight, and in terms of the onset of adverse gastrointestinal effects. In particular, long-acting agents appear to result in a greater reduction in HbA1c rates than short-acting agents [31].

### GLP-1 RA Effects on Atherogenesis and Oxidative Stress

Studies and experiments conducted on animals and human cells have shown that GLP-1 receptors expressed on endothelial cells, monocytes, macrophages and smooth muscle cells produce numerous effects that potentially interfere with the process of forming or rupturing atherosclerotic plaques [20].

We know that, at the basis of pathophysiological mechanisms of plaque formation, LDL cholesterol is transported through the intimal layer of the vessels and partly oxidized into oxidized LDL particles (oxLDL) through reactive oxygen species (ROS). Monocytes and macrophages that come into contact with oxLDL and ROS promote further infiltration of monocytes through the secretion of adhesion molecules such as VCAM-1, monocyte chemoattractive protein 1 (MCP-1), intercellular adhesion molecule 1 (ICAM-1) and E-selectin. OxLDL induces the transformation of monocytes into macrophages. These produce pro-inflammatory cytokines such as tumour necrosis factor alpha (TNF-α), interleukin (IL)-6 and IL-1b. They also incorporate lipid particles through phagocytosis and suppress the production of endothelial nitric oxide synthase (eNOS), leading to a reduction in NO-mediated vasodilation. In an environment dominated by ROS and oxLDL, macrophages are transformed into foam cells, which, by releasing their lipid content, contribute to the genesis or growth of atherosclerotic plaques. As an atherosclerotic plaque increases in size, necrotic areas form, endothelial cells (EC) undergo apoptosis and matrix metalloproteinases (MMPs) proteolytically destroy the fibrous cap. This results in plaque rupture, thrombus formation and bleeding in areas of the necrotic plaque. 

Recent studies have shown that GLP-1, along with exenatide, liraglutide and semaglutide, is able to reduce the production of ROS [20]. Stimulation of the GLP-1 receptor by reducing oxLDL production leads to a reduction in the activation of monocytes and macrophages and, consequently, of adhesion molecules [32,33,34,35]. This leads to a reduction in monocytes in the vascular wall and a greater expression of eNOS by ECs with an increased production of NO and a suppression of endothelin formation with consequent vasodilation [36]. Reduced exposure to ROS after stimulation of the GLP-1 receptor slows down the foam cell formation process and reduces their caspase-mediated apoptosis [37]. In confirmation of this hypothesis on oxidative stress, Lambadiari et al. demonstrated how the use of GLP-1RAs is able to reduce the levels of malondialdehyde (MDA) and thiobarbituric acid reactive substances (TBARS), metabolites deriving from highly reactive and unstable oxidative stress products [38]. Furthermore, stimulation of the GLP-1 receptor reduces the proliferation of vessel smooth muscle cells [39,40] and their possible migration into the plaque and induces the stabilization of the plaque itself [41,42].

## 3. Extra Virgin Olive Oil (EVOO)

Olive oils (OOs) are classified according to their quality into extra virgin olive oil (EVOO), virgin olive oil (VOO) and ordinary olive oil [43], and OO in its production variants of VOO and EVOO is universally recognized as a symbol of the MedDiet [44].

EVOO, obtained by mechanical extraction, is, simultaneously, the main source of fat and the main healthy component of the MedDiet; in fact, it reduces the risk of cancer, neurodegenerative diseases and metabolic diseases and also prevents the onset of cardio-cerebrovascular diseases [45,46,47,48,49]. EVOO is a virgin olive oil with a maximum free acidity, in terms of oleic acid, of 0.8 g of acid per 100 g of oil; VOO is a virgin olive oil with a maximum free acidity of 2 g per 100 g; ordinary olive oil is an olive oil with acidity not exceeding 3.3%.

There is abundant evidence indicating that the MedDiet possesses properties capable of attenuating the inflammatory response, especially in relation to chronic diseases. These properties have been attributed, above all, to the high intake of EVOO, which is the cornerstone of this diet [50]. This is due to a down-regulation of the expression of pro-inflammatory genes and low levels of pro-inflammatory proteins, but also to a lower plasma/serum concentration of pro-inflammatory markers, both in the postprandial and chronic phases [51].

### 3.1. EVOO and Inflammatory Markers

PREDIMED (Prevention with Mediterranean Diet), a randomized, multicentre primary prevention study, demonstrated how adherence to the MedDiet, rich in EVOO, leads to a reduction in pro-inflammatory interleukins, such as IL-6 and C-reactive protein (CRP) in subjects at high risk of cardiovascular disease [52]. It also showed how, in subjects with metabolic syndrome (MS), EVOO consumption is not only able to reduce the levels of pro-inflammatory interleukins (IL-6, IL-7, IL-8) and CRP, but also to improve endothelial function and insulin sensitivity [53]. After a median follow-up of 4.1 years, a statistically significant 40% reduction in the relative risk of new-onset T2DM was observed in the group that received the MedDiet supplemented with EVOO, but not in the group that received the MedDiet supplemented with nuts, compared to the control diet, suggesting a central role of EVOO in the prevention of diabetes [54]. 

Furthermore, recent meta-analyses of RCTs have consistently shown that the replacement of carbohydrates (5–10% of total energy intake) with MUFAs as a specific dietary compound has beneficial effects on metabolic risk factors in patients with T2DM [16,17]. In a meta-analysis of 32 cohort studies, in fact, MUFAs from EVOO were shown to produce a significant reduction in the risk of all causes of mortality, the onset of stroke and cardiovascular events [18]. In a systematic review analysing data from 4 cohort studies and 29 RCTs, the effects of diets enriched with EVOO on the risk of T2DM onset in healthy individuals and on the monitoring of glycaemic control parameters in patients already suffering from T2DM were investigated. It was found that consuming EVOO is associated with a reduced risk of developing T2DM and an improvement in glucose metabolism. These effects are not irrelevant if we consider that studies have shown that a 1% increase in HbA1c increases the risk of mortality from all causes by 28% [55].

Among the additional properties of EVOO, there is also its ability to prevent the down-regulation of adiponectin and increase the anti-inflammatory effect of HDL [56,57]. Moreover, Lorente-Cortes et al. reported the down-regulation of pro-thrombotic genes in addition to pro-inflammatory ones in subjects at high cardiovascular risk who followed an EVOO-enriched diet [58] (Table 1).

### 3.2. EVOO and Bioactive Compounds

EVOO has aroused particular interest for its beneficial and healthy potential due to its biological properties. In addition to having a high content of MUFAs, EVOO contains a number of “bioactive compounds”, including tocophenols, polyphenols, squalene, phytosterols and tocopherols (vitamin E).

The phenolic content of EVOO consists of: phenolic acids (e.g., caffeic, vanillic, coumaric, ferulic and sinapic acids), phenolic alcohols (e.g., tyrosol and hydroxytyrosol), secoiridoids (e.g., oleuropein), lignans (e.g., pinoresinol) and flavones (e.g., luteolin and apigenin) [59,60].

Initially, EVOO’s health benefits were attributed to its high MUFA content. Among these, oleic acid is the most important fat with effects on gastrointestinal and metabolic functions and on cardiovascular risk factors [61]. In fact, it has been stated that the consumption of oleic acid favours better control of the secretory activity of the pancreas and liver (biliary secretion), as well as better gastric mucosa protection through the reduced secretion of hydrochloric acid, which helps limit the risk of gastro-duodenal ulcers [62]. 

Tocopherols are essentially present as α-tocopherol, which is the main form of this vitamin in human tissues [63]. This is one of the most important naturally occurring lipophilic antioxidants [64,65], and its role in preventing lipid peroxidation of cell membranes and lipoproteins [66] has been recognized by the European Food Safety Authority (EFSA) [67]. Both the redox-dependent and independent properties of this vitamin have been shown to influence the expression of homeostatic genes that protect tissues from oxidative and inflammatory processes associated with aging, degenerative diseases and cancer [63].

Marrano et al. investigated the effects of different phenolic compounds (PCs) on beta cell function and survival. Survival, insulin biosynthesis, glucose-stimulated insulin secretion (GSIS) and activation of intracellular signalling were evaluated. Hydroxytyrosol, tyrosol and apigenin increased the proliferation of beta cells and the biosynthesis of insulin, promoting the health of these cells. Apigenin and luteolin enhanced GSIS. In contrast, vanillic acid and vanillin were pro-apoptotic for beta cells, although they increased GSIS. Apigenin has been shown to be the most effective compound and also the one capable of activating intracellular signalling. Therefore, it was concluded that EVOO, thanks to these compounds, could improve insulin secretion and promote glycaemic control in T2DM patients [68].

### 3.3. EVOO and Atherosclerosis

Among EVOO’s positive health effects, one of the most studied has been its antioxidant power and the link between this and atherosclerotic diseases [69]. Atherosclerosis is a pathological condition characterized by a cascade of events that occur mainly in the vascular wall, such as endothelial activation, the infiltration of macrophages and, finally, the formation of plaques [70]. 

In this context, it is important to remember the effects of EVOO on endothelial function both in the fasting and postprandial phases. EVOO consumption, in fact, improves endothelium functionality and, in particular, the vasodilator capacity during the postprandial phase [71]. Two of the methods used to quantify endothelial damage are the estimation of microparticles released into the circulation and the determination of endothelial progenitor cells, which reflects the ability of the endothelium to regenerate. A study has shown that the chronic use of EVOO in the diet is related to a lower number of microparticles found in the circulation and a higher level of endothelial progenitor cells than those observed in other dietary regimes. These effects were also accompanied by a lower concentration of NO degradation products in the urine, indicating a reduction in oxidative stress [72].

An in vitro study investigated the effect of EVOO on ECs in relation to stimuli induced by vascular endothelial growth factor (VEGF). EVOO, and in particular its phenolic component, significantly reduced the VEGF-induced migration of cells, as well as reduced ROS through the modulation of NADPH oxidase (Nox) [73].

Polyphenols may affect glucose metabolism at various levels: through an inhibition of digestion and absorption of carbohydrates, a reduction in the release of glucose from the liver or a stimulation of glucose uptake in peripheral tissues [74]. With their antioxidant properties, polyphenols could decrease the production of advanced glycosylated end products such as HbA1c [75]. The polyphenols contained in EVOO play a role in the activation of Nrf-2 in the liver and in the consequent release of antioxidant enzymes [76]. Indeed, Nrf-2 is believed to be the main regulator of redox homeostasis, and its activation inhibits pro-inflammatory mediators such as cytokines, COX-2 and iNOS [77]. EVOO polyphenols limit inflammation by reducing the expression of the transcription factors NF-κB and AP-1 thanks to their ability to scavenge free radicals and break the radical chain with a reduced formation of ROS and of reactive nitrogen species (RNS) [78]. In this context, ROS play a key role in various signalling pathways that promote vascular inflammation in atherogenesis, from the beginning of the development of the lipid stria to plaque rupture [79]. LDL oxidation is certainly a crucial stage in the progression of atherosclerosis. Furthermore, oxLDL itself represents a powerful stimulus for the formation of ROS, thus contributing to the inflammatory state that characterizes atherosclerosis [80]. Nox and, in particular, its Nox-2 isoform, a key enzyme in the process of atherogenesis, represents one of the enzymatic pathways capable of producing ROS in the vessel wall [81]. Therefore, considering the role of oxidative stress in the pathogenesis of atherosclerosis, the antioxidant effect of the phenolic compounds contained in EVOO, which determine both a direct reduction in ROS and in the Nox-2 activation marker (sNox-2-dp serum), has been shown [82,83]. The improvement in glycaemic and lipid homeostasis given by these EVOO properties prevents the onset of chronic diseases, including MS.

The MedDiet with the addition of EVOO brings benefits to the postprandial profile by reducing blood sugar, LDL cholesterol and oxLDL and by increasing circulating insulin levels in healthy subjects [84]; in addition, thanks to the properties of EVOO, it has been associated with both a reduction in atherogenic LDL-C cholesterol and levels of non-high-density (non-HDL-C) lipoprotein cholesterol [85]. Covas et al. showed that the intake of the phenolic compounds contained in EVOO increases HDL-C, and decreases the ratios of TC/HDL-C, LDL-C/HDL-C and triglycerides [86].

## 4. EVOO, GLP-1 and Glycaemic Control Improvement: Possible Underlying Mechanisms

**Figure 1:**
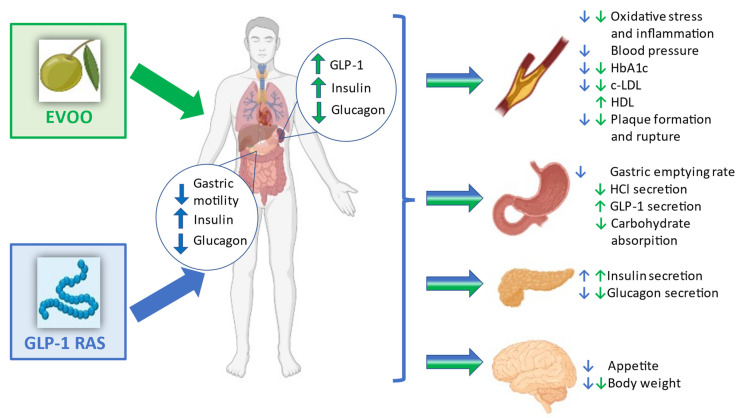
EVOO and GLP-1 RA actions on gastric and pancreatic functions and their possible synergistic effects on glycaemic control and cardiovascular risk. The green arrows symbolize EVOO actions, the light blue, GLP-1 RA actions.

The positive effects of EVOO are related to the up-regulation of incretins as it reduces the activity of DPP-4, resulting in an increase in the concentration of GLP-1, which modulates postprandial blood glucose through insulin secretion [87] (see Figure 1).

Another possible explanation of the link between EVOO, GLP-1 and the consequent glycaemic control, from a mechanistic point of view, derives from the study by Garcia-Serrano et al., which showed that the intake of EVOO produced favourable changes in GLP1 levels, resulting in greater postprandial GLP-1 concentrations when compared with olive oil and sunflower oil [88].

Other interesting data come from studies on the levels of circulating lipopolysaccharides (LPS) in patients who do not have sepsis and which may be an indicator of cardiovascular risk. In fact, high levels can be found in obese patients with T2DM or in patients presenting with an acute cardiovascular event [89,90].

In T2DM, the term “metabolic endotoxemia” is used to describe the association between low-grade endotoxemia and metabolic changes such as insulin resistance, hyperglycaemia and alterations in lipid metabolism that promote obesity [91]. This occurs as a result of changes in intestinal permeability involving translocation of the circulating LPS [92]. This explains how changes in intestinal permeability can positively affect glycaemic control in T2DM [93].

**Table 1:** Features of the studies published on EVOO and MedDiet beneficial effects.

Author/Year	Design of the Study	Drug	Outcome(s)	Conclusion
**Fuentes et al. [71], 2001**	Intervention dietary study Patients n°: 22	Low-saturated fat diet vs. MedDiet	Determine endothelial function in hyper-cholesterolemic patients in the two different diets	Flow-mediated dilatation increased during the MedDiet levels of plasma cholesterol, LDL, ApoB and P-selectin decreased with both diets
**Esposito et al. [53], 2004**	Randomized, single-blind trial Patients n°: 180 with metabolic syndrome (MetS)	MedDiet vs. Control diet	Nutrient intake endothelial function score lipid and glucose parameters, insulin sensitivity and circulating levels of hs-CRP and interleukins 6 (IL-6), 7 (IL-7) and 18 (IL-18).	Body weight and hs-CRP decreased Endothelial function score improved The number of patients with metabolic syndrome decreased
**Mitjavila et al. [83], 2012**	Randomized, controlled, trial Patients n°: 110 with MetS	MedDiet + EVOO 1Lt/week or MedDiet + mixed nuts vs. Low-fat diet	Test the efficacy of MedDiet on the primary prevention of cardiovascular diseases	MedDiet reduces oxidative damage to lipids and DNA in MetS individuals
**Loued et al. [57], 2013**	Interventional study Patients n°: 20 healthy subjects (divided in two groups: 10 young and 10 elderly)	EVOO 25 mL/day administered to the two groups	Investigate the effect of ageing and the role of PON1 on the anti-inflammatory activity of HDL Determine whether EVOO consumption could improve the atheroprotective activity of HDL	EVOO consumption increased the anti-inflammatory activities of both HDL and PON1 The anti-inflammatory activity of HDL was modulated by PON1 and was lower in the elderly volunteers EVOO consumption increased the anti-inflammatory effect of HDL and reduced the age-related decrease in anti-atherogenic activity
**Carnevale et al. [82], 2014**	Randomized controlled trial Patients n°: 25 healthy subjects	MedDiet with EVOO 10 gr/day vs. MedDiet without EVOO	Investigate the role of EVOO in the atherosclerotic process	Addition of EVOO to a MedDiet protects against postprandial oxidative stress
**Salas-Salvadó et al. [54], 2014**	Randomized controlled trial Patients n°: 3541 without diabetes at high cardiovascular risk	MedDiet supplemented with EVOO vs. MedDiet supplemented with nuts vs. Control diet (advice on a low-fat diet)	Assess the efficacy of MedDiet for the primary prevention of diabetes	MedDiet supplemented with EVOO reduced diabetes risk among persons with high cardiovascular risk
**Violi et al. [84], 2015**	Interventional cross-over study Patients n°: 25 healthy subjects	MedDiet with EVOO 10 gr/day vs. MedDiet without EVOO	Find the mechanisms that make EVOO effective in the prevention of cardiovascular disease	Decrease in blood glucose, DPP-4 protein and activity, LDL-C, oxLDL Increase in insulin, GLP-1, GIP
**Santangelo et al. [55], 2016**	Interventional study Patients n°: 11 with T2DM, overweight but non-insulin treated	Abutal diet supplemented with EVOO 25 mL/day	Improvement in anthropometric parameters, fasting glycaemia, HbA1c, high-sensitive CRP, plasma lipid profile, liver function and serum levels of TNF-α, IL-6, adiponectin, visfatin.	EVOO significantly reduced fasting plasma glucose, HbA1c, BMI, and body weight, serum levels of AST and ALT and serum visfatin levels
**Carnevale et al. [87], 2017**	Interventional cross-over study Patients n°: 30 with IFG	Meal with 10 gr of EVOO vs. Meal without EVOO	Improvement in postprandial glycaemia	EVOO reduces glycemia and DPP-4 activity Increases insulin and GLP-1 and decreases triglycerides and Apo B
**Marrano et al. [68], 2021**	In vitro study	INS-1E cells were exposed to 10 μM of the main EVOO PCs for up to 24 h	To investigate the effects of several phenolic compounds (PCs) on beta-cell function and survival	EVOO may improve insulin secretion and promote glycaemic control in T2DM patients
**Bartimoccia et al. [93], 2022**	Interventional study Patients n°: 20 with IFG and 20 healthy subjects	Mediterranean-type meal with 10 gr of EVOO vs. Mediterranean-type meal without EVOO	Improvement in postprandial glycaemia by reducing gut permeability-derived low-grade endotoxemia	IFG patients assuming EVOO showed a less significant increase in blood glucose, blood insulin and GLP1 and a significant reduction in LPS and zonulin compared to IFG patients not given EVOO

Apolipoprotein B (ApoB); dipeptidyl peptidase-4 (DPP-4); extra virgin olive oil (EVOO); glucagon-like peptide 1 (GLP1); high-sensitivity C-reactive protein (hs-CRP); impaired fasting glucose (IFG); Mediterranean diet (MedDiet); metabolic syndrome (MetS); paraoxonase 1(PON1); type 2 diabetes mellitus (T2DM).

Studies conducted by Bartimoccia et al. on populations of diabetic patients have shown how taking EVOO or its oleuropein component is able to reduce postprandial glycaemic and LPS levels whose increase determines higher cardiovascular risk [94,95,96,97]. Moreover, Bartimoccia et al. showed that the administration of 10 g/day of EVOO, in addition to reducing postprandial glycaemia in patients with impaired fasting glucose (IFG), increases blood insulin and GLP-1 levels; conversely, a reduction in LPS and zonulin (a marker of intestinal permeability) compared to IFG patients not treated with EVOO was observed [93].

Another study conducted by Carnevale et al. demonstrated, in a group of IFG patients, that taking EVOO with meals results in an increase in circulating levels of GLP-1 and in insulinemia, in the postprandial phase, of about 40% in comparison with the IFG patient population not receiving EVOO. They also recorded a reduction in blood glucose levels of about 20% compared to controls, as well as a reduction in DPP-4 levels. The postprandial lipid profile was also improved in patients consuming EVOO; in particular, a reduction in ApoB-48 was recorded. This could be explained with an increase in circulating GLP-1, which would have a role in the down-regulation of ApoB-48 itself [87].

The release of intestinal hormones, GLP-1 incretin hormones and glucose-dependent insulinotropic polypeptide (GIP) can be mediated by various dietary fats [98,99]. During their digestion in the small intestine, fatty acids and 2-monoacylglycerol are generated from these [100]. This digestive process is important as it stimulates the secretion of incretin hormones [101] whose release is not dependent on calories but on the molecules ingested in the meal.

Mette et al. showed that the intake of 20 mL of EVOO induces an increase in plasma concentrations of GLP-1 as well as a greater release of GIP compared to the other study groups. There was also a reduction in plasma glucose response, which could be due to a combined effect of increased concentrations of insulin, incretin hormones and cholecystokinin, known to delay stomach emptying [102].

## 5. Conclusions

The rising burden of T2DM is becoming an urgent public health concern, and efficient therapeutic approaches as well as preventive measures are needed to avoid mortality and morbidity consequences. Among these, changes in lifestyle in terms of the reduction in unhealthy diets and the choice of an EVOO-enriched MedDiet could be a first step, with the prospect of making incretin-based therapies more effective, where indicated, according to international guidelines.
